# Tuberculosis under the Influence of COVID-19 Lockdowns: Lessons from Tehran, Iran

**DOI:** 10.1128/mSphere.00076-21

**Published:** 2021-02-24

**Authors:** Mansour Kargarpour Kamakoli, Shima Hadifar, Sharareh Khanipour, Ghazaleh Farmanfarmaei, Abolfazl Fateh, Shayan Mostafaei, Seyed Davar Siadat, Farzam Vaziri

**Affiliations:** a Department of Mycobacteriology and Pulmonary Research, Pasteur Institute of Iran, Tehran, Iran; b Microbiology Research Center (MRC), Pasteur Institute of Iran, Tehran, Iran; c Clinical Research Development Center, Imam Reza Hospital, Kermanshah, Iran; d Epidemiology and Biostatistics Unit, Rheumatology Research Center, Tehran University of Medical Sciences, Tehran, Iran; University of Michigan-Ann Arbor

**Keywords:** tuberculosis, COVID-19, lockdowns, Iran

## Abstract

This study investigates the short-term effects of the coronavirus disease 2019 (COVID-19) pandemic lockdown on tracing and detection of tuberculosis (TB) patients in Tehran, Iran. Results of this study have demonstrated that due to the significant decrease in the identification of patients with suspected TB during the COVID-19 outbreak in Tehran, it is imperative that patients with suspected TB be tracked and diagnosed more quickly to make up for some of the decline in TB diagnosis in recent months and to recover lost cases.

## PERSPECTIVE

Tuberculosis (TB) remains the top cause of global mortality from infectious diseases with an estimated 1.4 million deaths in 2018 (1). In January 2020, the emergence of SARS-CoV-2 as a pandemic attracted global attention and was seen as a priority for all governments. This situation along with strict lockdowns imposed to control the pandemic has had disruptive effects on the regional routine TB services and the national program for TB elimination (2–4). Accordingly, in this study, we sought to investigate the short-term effects of the coronavirus disease 2019 (COVID-19) pandemic lockdown on tracing and detection of TB patients in Tehran, Iran.

## STATISTICAL METHODS

Categorical variables are presented as *n* (%). Time trend curves were tested by linear trend test (Z-test statistic). Also, linear slopes and 95% confidence intervals (95% CIs) were estimated based on the nonparametric trend tests done by “sens.slope” function in the “trend” R package. Moreover, polynomial slopes and *R*^2^ for nonlinear trend curves were calculated using a robust local polynomial trend model. The patterns of data were identified using LOWESS smoother. A two-sided *P* value of less than 0.05 was considered statistical significance.

### Biomedical and socioeconomic impacts of the COVID-19 lockdowns on tuberculosis.

**FIG 1:**
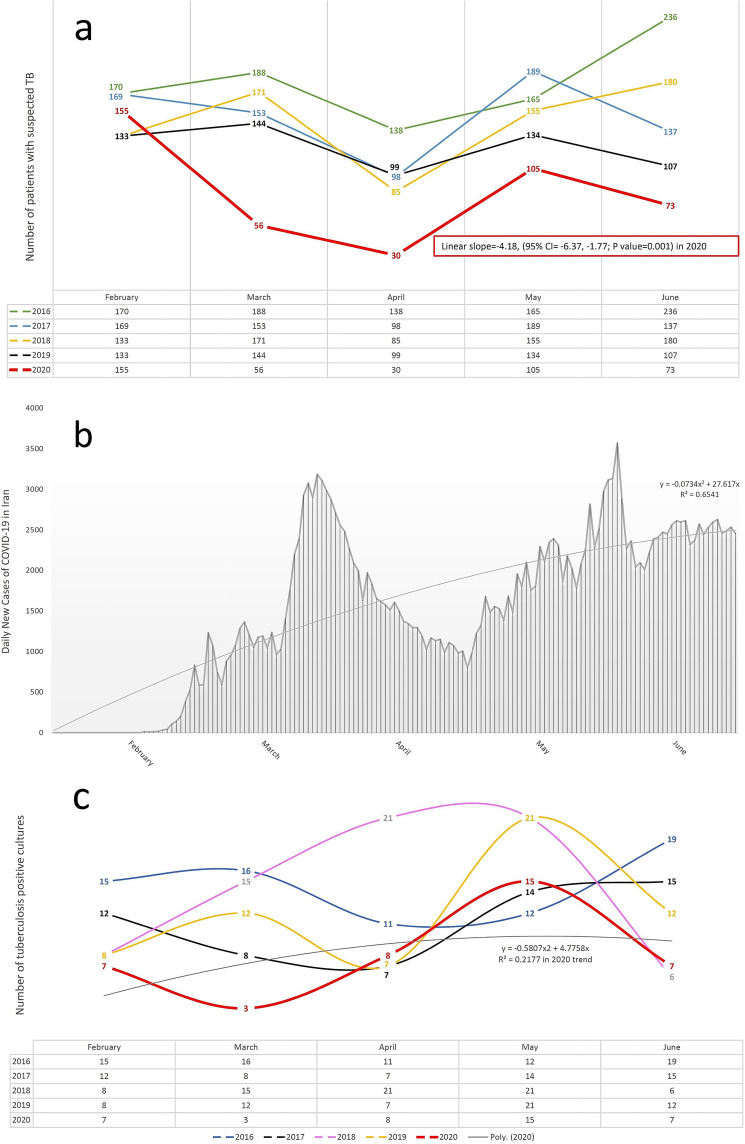
Changes in frequency of suspected TB patients, TB-positive cultures, and COVID-19 patients. (a) Frequency of suspected TB patients who were registered at the Department of Mycobacteriology and Pulmonary Research, Pasteur Institute of Iran, between 2016 and 2020 (February to June in each year). (b) Daily number of COVID-19 patients in Iran. (c) Number of TB-positive cultures between February and June, 2016 to 2020. Poly. (2020), polynomial trend curve (2020).

The occurrence of COVID-19 adversely affected TB incidence and deaths. The consequences of lockdowns, impaired detection of new cases of TB, and dormant TB reactivation as a result of the viral pandemic can be reasons for this situation to occur (2, 5). India has recently shown a 59% decline in the diagnosis of TB in 8 weeks of lockdown (6). It was also predicted worldwide that due to a 25% reduction in the diagnosis of TB in a 3-month period of lockdown, 190,000 additional deaths due to TB would occur in 2020 (3). Interestingly, the decline in multidrug resistance (MDR) TB screening in China occurred at the time of the COVID-19 outbreak (7). Another report also predicted that due to lockdown, 4% of surplus deaths in the world and 5.7% of surplus deaths in India will happen due to TB between 2020 and 2025 (4). Also, a recent study in Italy showed that despite efforts to maintain TB services, these services were significantly disrupted during the COVID-19 outbreak (8). Our short-term study of the statistics of patients with suspected TB during the COVID-19 outbreak and the resulting lockdown in Iran also confirms these results and predictions. Our review shows that the number of patients with suspected TB in March and April 2020 (simultaneously with the outbreak of the COVID-19 epidemic in Iran) had a sharp decrease compared to previous years (65.9% and 71.4% decrease, respectively). In other words, the linear slope of the suspected TB patient trend curve in 2020 was −4.18 (95% CI = −6.37, −1.77; *P* value = 0.001). Also, in 2016, 2018, and 2019, there was an increase in the number of patients with suspected TB in March compared to February, but in 2020, we saw a sharp decrease in the number of patients with suspected TB in March compared to February. In all months of the outbreak of COVID-19 in Iran (from March to June 2020), the lowest number of patients with suspected TB was recorded (55.6% decrease) compared to previous years (2016 to 2020) (Fig. 1a).

These results were obtained in a situation where March and April were part of the period of lockdown and closure of jobs in Iran, because the number of patients with COVID-19 peaked in March and April (9), many businesses closed in those 2 months, and most people tried to lock themselves down. At the end of April, the number of COVID-19 patients per day decreased. But most of the jobs in Iran resumed in May and lockdowns were lifted, which led to a reincrease in the number of people with COVID-19 in Iran from May onwards. The polynomial slope of the COVID-19 time trend curve was (−0.073 × time^2^ + 27.61 × time, *R*^2^ = 0.65) (Fig. 1b).

Also, the number of TB-positive cultures in March 2020 was the lowest compared to the same month in all previous years (76.5% decrease). The polynomial slope of the TB-positive culture time trend curve was (−0.58 × time^2^ + 4.77 × time, *R*^2^ = 0.217) in 2020 (Fig. 1c).

All of these statistics reflect the fact that lockdowns due to COVID-19 had a significant impact on the identification of TB patients in this short period.

Decreased diagnosis of TB can be due to the lockdown and the fear of visiting health centers (to diagnose TB) during COVID-19 lockdown (2). On the other hand, our data show that in all months (even after the lockdown was lifted in May) of the outbreak of the COVID-19 epidemic in Iran (from March to June 2020), the lowest number of patients with suspected TB (55.6% decrease) was recorded compared to previous years (2016 to 2020). This can be caused by the fear of going to health centers (to diagnose TB) throughout the COVID-19 outbreak.

Perhaps another reason for the decrease in patients with suspected TB in Iran (simultaneously with the outbreak of the COVID-19 epidemic) is the similarity of the symptoms of COVID-19 and TB. People with these two diseases show similar clinical manifestations such as fever, cough, and respiratory problems, and both diseases initially affect the lungs (10). Thus, perhaps in this pandemic period most people focus only on COVID-19, and after such symptoms occur, they suspect only COVID-19 and seek home treatment or visit COVID-19 diagnostic centers. This can be even more frustrating when a person has the two diseases simultaneously, because predictions show that people with both TB and COVID-19 can have poorer treatment outcomes, especially if TB treatment is disrupted (10). Furthermore, people who have a current TB infection or a history of TB are more likely to have more severe consequences after a COVID-19 infection due to lung damage (4, 11, 12). Therefore, TB should not be overlooked during the outbreak of COVID-19.

Another problem that has arisen during the COVID-19 lockdown has been the increase in unemployment and poverty in a significant part of Iranian society. In this regard, the Iranian Ministry of Labor has announced that 783,000 people have complained of unemployment since 13 March 2020. A government spokesman also said that 4 million jobs are at risk of being lost as a result of the COVID-19 outbreak and that 10 business groups were closed as soon as the virus was confirmed (13). These will cause poverty in Iranian society, and poverty is directly related to malnutrition. Malnutrition causes TB to progress to active TB because poor nutrition impairs innate and adaptive immunity (14). It has also been recently shown that about half of people with TB were poor (15). Other studies have also shown that reduced protein intake in poor people increases their risk of developing TB (16, 17). Thus, lockdowns due to COVID-19 can increase TB in the long term by increasing poverty, unemployment, and malnutrition.

## CONCLUSION

Due to the significant decrease in the identification of patients with suspected TB during the COVID-19 outbreak in Tehran, it is imperative that patients with suspected TB be tracked and diagnosed more quickly to make up for some of the decline in TB diagnosis in recent months, to recover lost cases, and also to mitigate the impact of COVID-19 on routine TB services and TB incidence and mortality. Lockdowns should also be managed in such a way that a dangerous infectious disease such as TB is not ignored.
